# Non-invasive PECS model for detection of combined post-capillary pulmonary hypertension

**DOI:** 10.3389/fmed.2025.1660387

**Published:** 2025-10-22

**Authors:** Chen-Yu Wan, Yu-Xuan Liu, Su-Gang Gong, Qin-Hua Zhao, Ci-Jun Luo, Hong-Lin Qiu, Wen-Hui Wu, Yun-Bo Wei, Lei Du, Ming-Feng Gu, Lan Wang, Rong Jiang, Ze-Pu Li

**Affiliations:** ^1^Tongji University School of Medicine, Shanghai, China; ^2^Department of Cardio-Pulmonary Circulation, Shanghai Pulmonary Hospital, Tongji University School of Medicine, Shanghai, China; ^3^Department of Cardiology, Affiliated Renhe Hospital of Shanghai University (Renhe Hospital, Baoshan District), Shanghai, China; ^4^Department of Cardiology, Shanghai General Hospital, Shanghai Jiao Tong University School of Medicine, Shanghai, China

**Keywords:** pulmonary hypertension, combined post-Capillary pulmonary hypertension, non-invasive diagnostics, echocardiography, right heart catheterization

## Abstract

**Background:**

Combined post-capillary pulmonary hypertension (Cpc-PH) is a severe form of pulmonary hypertension associated with high morbidity and mortality. Early identification and intervention are crucial but challenging due to the invasive right heart catheterization (RHC). This study aimed to develop and validate a non-invasive diagnostic model, the Predictive Echocardiography Cpc-PH Score (PECS), using echocardiographic parameters to facilitate detection of Cpc-PH.

**Methods:**

A retrospective analysis encompassing 198 patients with suspected PH-LHD, admitted from July 2010 through December 2023, was executed. Patients were divided into Cpc-PH and Ipc-PH/No-PH groups based on RHC in accordance with the 7th World Symposium on Pulmonary Hypertension criteria for PECS model construction. Chi-square and L1-regularized backward elimination refined predictive indicators. Model efficacy and stability were appraised via receiver operating characteristic and 5-fold cross-validation.

**Results:**

The PECS model, incorporating a suite of indicators including valvular heart disease, left atrial systolic diameter, interventricular septal thickness, mitral valve E/Em ratio, left ventricular fractional shortening, and tricuspid regurgitation velocity, demonstrated good predictive performance, achieving an area under characteristic (AUC) of 0.761 (95% CI: 0.692–0.823, *P* < 0.001). It demonstrated a sensitivity of 66.7%, specificity of 72.0%, a positive predictive value of 72.9%, a negative predictive value of 65.7%, and an overall accuracy of 69.2%. A total of 5-fold cross-validation confirmed these findings, yielding an AUC of 0.752 ± 0.070.

**Conclusion:**

The PECS model provides a non-invasive and precise approach to diagnosing Cpc-PH, potentially acting as a practical screening tool.

## Introduction

Post-capillary pulmonary hypertension (pcPH) represents a significant clinical challenge, particularly in combined post-capillary PH (Cpc-PH), which is associated with a poorer prognosis. Prompt identification and intervention are crucial for effective management (1, 2). The distinction between isolated post-capillary PH (Ipc-PH) and Cpc-PH has been emphasized in the evolving guidelines set forth by the European Society of Cardiology (ESC) and the European Respiratory Society (ERS) (3, 4). According to the 2015 ESC/ERS guidelines, Cpc-PH is defined by specific hemodynamic criteria, including a mean pulmonary arterial pressure (mPAP) exceeding 25 mmHg, a pulmonary arterial wedge pressure (PAWP) greater than 15 mmHg, a diastolic pressure gradient (DPG) over 7 mmHg, or a pulmonary vascular resistance (PVR) exceeding three Wood units (WU) (1, 5–7). Recent updates in the 2022 guidelines and the 7th World Symposium on Pulmonary Hypertension (WSPH) have further refined these diagnostic thresholds, notably lowering the mPAP and PVR criterion to greater than 20 mmHg and 2 WU, respectively (1).

Given the invasive nature of right heart catheterization (RHC) (8), traditionally employed to confirm the diagnosis of Cpc-PH, there is a pressing need for non-invasive diagnostic alternatives. Heart failure (HF) patients, especially those with PH, often present with advanced age, frailty, and multiple comorbidities (9).

Echocardiography, a non-invasive diagnostic tool, has demonstrated great potential in screening and predicting PH due to its convenience, and cost-effectiveness (10). However, studies utilizing a PVR cutoff of 3 WU have shown limitations in the accuracy of echocardiography when predicting Cpc-PH (11). Furthermore, the ability of echocardiography to predict Cpc-PH in HF patients under the 7th WSPH criterion has not been fully investigated.

This study aims to address these critical gaps by establishing the Predictive Echocardiography Cpc-PH Score (PECS), providing clinicians with an improved non-invasive framework for therapeutic decision-making.

## Materials and methods

### Study population

This study retrospectively enrolled patients suspected of having pcPH at Shanghai Pulmonary Hospital between July 2010 and December 2023. All patients received a thorough evaluation by experienced clinicians and underwent RHC and echocardiography. Both examinations were completed during the same hospitalization, with an interval of no more than 7 days between transthoracic echocardiography and RHC, and no hemodynamically relevant therapies were initiated or modified in this period. The classification of Cpc-PH was based on the 7th WSPH criterion. This criterion includes an mPAP > 20 mmHg and a PAWP > 15 mmHg. In this framework, a PVR greater than 2 WU classifies the condition as Cpc-PH, while a PVR less than or equal to 2 WU designates it as Ipc-PH (1). To compare the 2015 ESC/ERS and 7th WSPH criteria, patients with Cpc-PH who met the 2015 ESC/ERS criteria (mPAP > 25 mmHg, PAWP > 15 mmHg, and PVR > 3 WU) were also identified (12). Other forms of PH (Group 1, Group 3, Group 4, and Group 5 PH) were excluded from the study. All participants provided written informed consent, and the study was approved by the Ethics Committee of Shanghai Pulmonary Hospital (K16-317).

### Hemodynamic assessment

Right heart catheterization, deemed the gold standard for diagnosing and classifying Group 2 PH (13), was employed to obtain key hemodynamic parameters, including mPAP, right atrial pressure (RAP), and PAWP. Cardiac output (CO) was measured using the indirect Fick method, while cardiac index (CI) was calculated by dividing CO by body surface area. PVR was derived by calculating the difference between mPAP and PAWP divided by CO. Hemodynamic data were interpreted by investigators blinded to the echocardiographic results.

### Transthoracic echocardiography

The echocardiographic measurement methods used in this study were consistent with those in previous studies to assess cardiac structure and function (General Electric Company, United States, Vivid 7 Dimension system) (14).

To assess left heart remodeling, left atrial end-systolic diameter (LAD), left ventricular end-diastolic diameter (LVEDD), left ventricular end-systolic diameter (LVESD), interventricular septum (IVS) and left ventricular posterior wall thickness (LVPWT). Left heart systolic function was assessed by ventricular ejection fraction (LVEF), left ventricular fractional shortening (LVFS) and mitral valve systolic wave velocity (MV Sm) (9). Right ventricular remodeling was assessed through the right atrial transverse diameter (RATD), right atrial longitudinal diameter (RALD), right ventricular end-diastolic longitudinal dimension (RVEDLD), right ventricular end-diastolic transverse dimension (RVEDTD) and right ventricular free wall thickness (RVFWT) (15). Right ventricular systolic function was assessed as tricuspid annular plane systolic excursion (TAPSE), and right ventricular S’ wave TV-Sm (16). The RAP was evaluated semi-quantitatively by assessing the maximum inferior vena cava diameter and its collapsibility (16). Systolic pulmonary artery pressure (PASP) was calculated by the sum of RAP and the systolic pressure gradient, obtained from peak tricuspid regurgitation velocity (TR Vmax) using the simplified Bernoulli’s equation (17). The TAPSE/PASP ratio serves as a valuable indicator of the coupling between the right ventricle (RV) and pulmonary artery (18). The end-diastolic eccentricity index of the left ventricle (ENDSEI) was measured as the compression of the RV on left ventricle. All echocardiographic image acquisitions and interpretations were performed by sonographers who were blinded to patients’ clinical data and to the results of RHC.

### Statistical analysis

Categorical variables were summarized using counts and percentages [n (%)]. Continuous variables were assessed for normality using Shapiro-Wilk or Kolmogorov-Smirnov tests, after which they were categorized into normally distributed and skewed distributed variables. Normally distributed variables were described using mean ± standard deviation, while skewed variables were characterized using median (interquartile range).

Prior to model construction, continuous echocardiographic parameters were dichotomized using the Youden Index to minimize the potential impact of multicollinearity among variables (19). Chi-square tests were conducted on medical history data and dichotomized echocardiographic parameters, with a significance level set at *P* < 0.1, to retain as many candidate variables related to Cpc-PH diagnosis as possible (20).

During the construction of the multivariate logistic regression model, L2 regularization (with a regularization coefficient C = 1) was initially employed to screen out variables with regression coefficients close to zero (21). Subsequently, the stepwise backward method was utilized to further optimize the model by eliminating the variable with the highest *P*-value that was not statistically significant until all variables in the model had *P*-values < 0.1. The regression coefficients of the final model variables were retained to one decimal place to serve as weights for the PECS model. The model’s performance was evaluated using the receiver operating characteristic (ROC) curve and the area under the curve (AUC) with corresponding 95% confidence intervals (22). The optimal cutoff value was determined based on the maximum Youden Index, and sensitivity, specificity, positive predictive value (PPV), negative predictive value (NPV), and overall accuracy were calculated to comprehensively reflect the model’s diagnostic discrimination ability. We further evaluated the model’s performance with 5-fold cross-validation. The dataset was randomly divided into five equal parts; in each fold, four-fifths of the data were used for training and one-fifth for validation (23). The AUC, sensitivity, and specificity were calculated for each fold, and their average values were reported to assess model generalizability. Missing data were minimal and were imputed by linear interpolation (24). Finally, to facilitate clinical application, a nomogram based on the final model was constructed to allow easy estimation of an individual patient’s Cpc-PH risk.

All statistical analyses were conducted using IBM SPSS Statistics for Windows (Version 29.0.2.0, IBM Corp., Armonk, NY, United States) and Python version 3.9.

## Results

### Demographic characteristics of the derivation cohort

**FIGURE 1:**
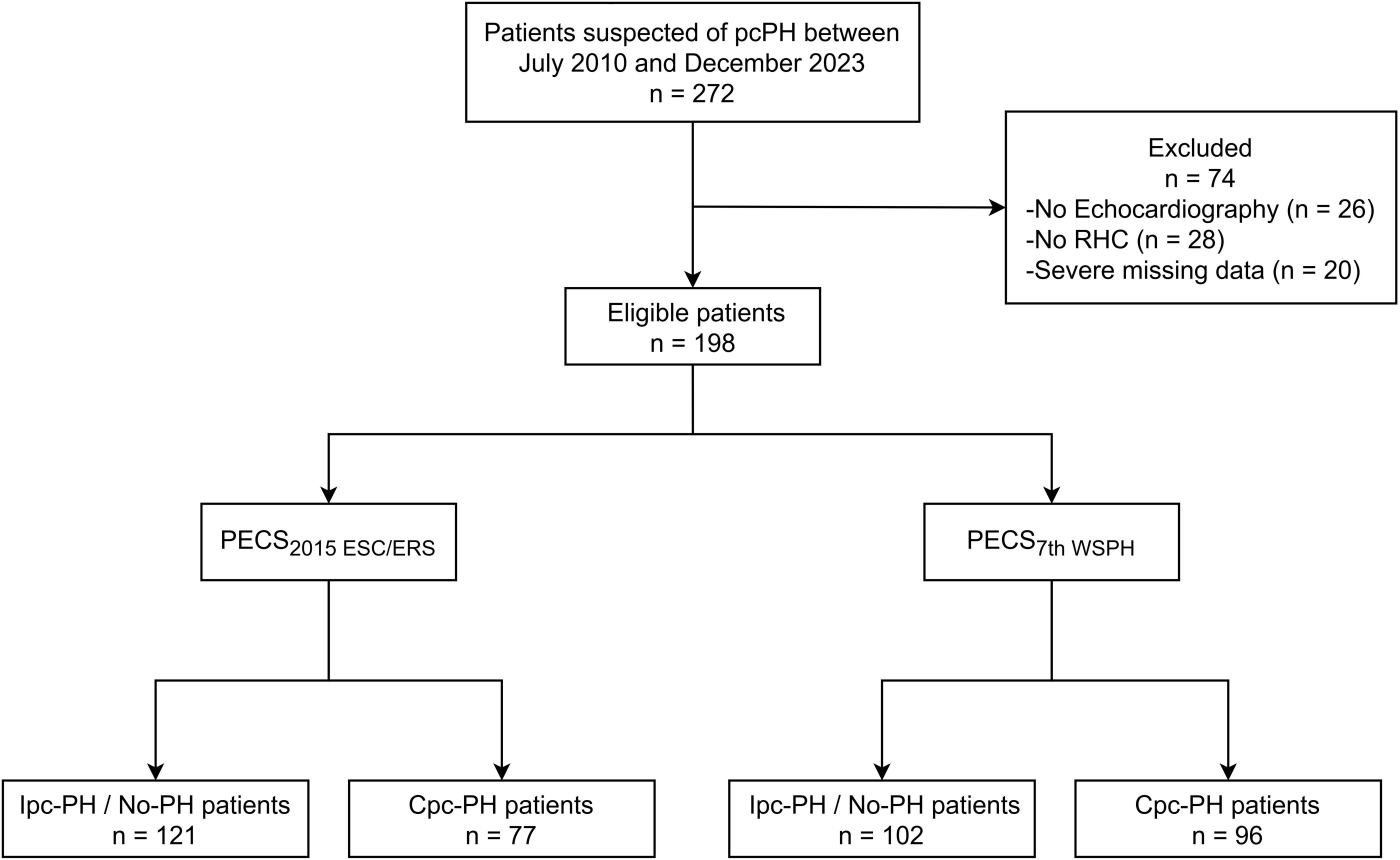
Flow diagram for the main derivation cohort. RHC, right heart catheterization; PECS, Predictive Echocardiography Cpc-PH Score; Ipc-PH, isolated post-capillary pulmonary hypertension; Cpc-PH, combined post-capillary pulmonary hypertension.

A total of 198 patients were enrolled in the derivation cohort (Figure 1). As this was a retrospective study, all 198 consecutive eligible patients were included, and the sample size was therefore not derived from a formal *a priori* power calculation. According to the 7th WSPH criteria, the cohort included 105 patients with Cpc-PH and 93 patients classified as Ipc-PH or No-PH patients. No significant differences were observed between the two groups with regard to demographic characteristics, diagnostic basis, or etiological distribution. No procedure-related adverse events were observed during either the echocardiographic examinations or the right-heart-catheterization procedures. However, Cpc-PH patients exhibited a higher prevalence of valvular heart disease (VHD) and mitral regurgitation, but a lower prevalence of bronchial asthma. Hemodynamic assessments revealed that Cpc-PH patients had higher RAP, mPAP, and PVR, whereas CO and CI were relatively lower (Table 1).

**TABLE 1:** Demographic characteristics and hemodynamic parameters of patients based on the 7th World Symposium on Pulmonary Hypertension (WSPH) criteria vs. 2015 European Society of Cardiology/European Respiratory Society (ESC/ERS) criteria.

Variable	2015 ESC/ERS criteria		7th WSPH criteria	
	Ipc-PH/No-PH	Cpc-PH	Ipc-PH/No-PH	Cpc-PH
	(*n* = 136)	(*n* = 62)	(*n* = 93)	(*n* = 105)
Age, years	67.8 ± 11.2	62.5 ± 14.1	67.5 ± 12.0	64.9 ± 12.6
Gender, *n* (%)	69 (50.7)	35 (56.5)	48 (51.6)	56 (53.3)
**Classification of HF, *n* (%)**				
HFrEF	4 (2.9)	3 (4.8)	2 (2.2)	5 (4.8)
HFmrEF	9 (6.7)	4 (6.5)	5 (5.4)	8 (7.7)
HFpEF	123 (90.4)	55 (88.7)	86 (92.5)	92 (87.6)
Etiology, *n* (%)				
IHD	35 (25.7)	19 (30.7)	24 (25.8)	30 (28.6)
HCM	2 (1.5)	2 (3.2)	2 (2.2)	2 (1.9)
VHD	52 (38.2)	32 (51.6)*	30 (32.3)	54 (51.4)^***^
MR	9 (6.7)	8 (12.9)	4 (4.3)	13 (12.4)^**^
AS	8 (5.9)	3 (4.8)	3 (3.2)	8 (7.6)
**Comorbidity, *n* (%)**				
CAD	44 (32.4)	23 (37.1)	30 (32.3)	37 (35.2)
Hypertension	80 (58.8)	37 (59.7)	56 (60.2)	61 (58.1)
DM	38 (27.9)	14 (22.6)	26 (28.0)	26 (24.8)
CKD	29 (21.3)	11 (17.7)	20 (21.5)	20 (19.1)
COPD	55 (40.4)	25 (40.3)	35 (37.6)	45 (42.9)
BA	12 (8.8)	2 (3.2)	10 (10.8)	4 (3.8)*
ILD	2 (1.47)	2 (3.23)	2 (2.15)	2 (1.90)
PE	12 (8.9)	6 (9.7)	8 (8.6)	10 (9.5)
Hyperthyroid	11 (8.1)	9 (14.5)	9 (9.7)	11 (10.5)
OSAS	1 (0.7)	0 (0.0)	0 (0.0)	1 (1.0)
PMI	6 (4.4)	4 (6.5)	5 (5.4)	5 (4.8)
AF	70 (51.5)	26 (41.9)	44 (47.3)	52 (49.5)
Dyslipidemia	23 (16.9)	12 (19.4)	17 (18.3)	18 (17.1)
Anemia	20 (14.7)	7 (11.3)	16 (17.2)	11 (10.5)
**Right heart catheterization**				
HR, bpm	78.9 ± 16.2	83.4 ± 17.4	80.2 ± 16.5	80.4 ± 16.9
SBP, mmHg	153.7 ± 30.4	150.2 ± 29.2	151.8 ± 28.2	153.3 ± 31.7
DBP, mmHg	79.4 ± 14.0	81.1 ± 18.0	79.2 ± 14.3	80.6 ± 16.2
RAP, mmHg	7.0 (4.0, 10.0)	8.0 (5.0, 11.0)	7.0 (4.8, 9.2)	8.0 (5.0, 12.0)
sPAP, mmHg	48.5 (40.8, 59.0)	71.0 (61.2, 87.0)	47.0 (38.0, 56.0)	64.0 (54.0, 82.0)
dPAP, mmHg	16.0 (13.0, 20.2)	23.0 (20.0, 31.0)	15.0 (12.0, 18.0)	21.0 (18.0, 26.0)
mPAP, mmHg	30.0 (25.0, 34.0)	42.0 (37.2, 51.5)	28.0 (24.0, 32.0)	38.0 (33.0, 45.0)
PAWP, mmHg	17.0 (14.0, 19.2)	21.5 (17.0, 25.0)	15.0 (13.0, 18.0)	20.0 (17.0, 24.0)
CO, L/min	5.4 (4.4, 6.2)	4.4 (3.8, 5.4)	5.4 (4.4, 6.2)	4.8 (4.0, 5.9)
CI, L/(min^⋅^m^2^)	3.2 (2.6, 3.7)	2.6 (2.2, 3.1)	3.2 (2.6. 3.7)	2.8 (2.3, 3.2)
PVR, WU	2.1 (1.6, 2.8)	4.6 (3.6, 5.7)	1.9 (1.4, 3.1)	3.3 (2.6, 4.9)

Values are expressed as the mean ± SD or median (Interquartile range). AS, aortic stenosis; BA, bronchial asthma; COPD, chronic obstructive pulmonary disease; CAD, coronary arterial disease; CI, cardiac index; CKD chronic kidney disease; CVD, cardiovascular disease; DBP, diastolic blood pressure; DM, diabetes mellitus; dPAP, diastolic pulmonary artery pressure; HCM, hypertrophic cardiomyopathy; HF, heart failure; HFpEF, heart failure with preserved ejection fraction; HFrEF, heart failure with reduced ejection fraction; IHD, ischemic heart disease; ILD, interstitial lung disease; mPAP, mean pulmonary artery pressure; MR, mitral regurgitation; OSAS, obstructive sleep apnea syndrome; PE, pulmonary embolism; PH, pulmonary hypertension; PMI, pacemaker implantation; PVR, pulmonary vascular resistance; SBP, systolic blood pressure; VHD, valvular heart disease.

*P < 0.1,

**P < 0.05,

***P < 0.01.

In accordance with the 2015 ESC/ERS criteria, the cohort included 136 patients with Cpc-PH and 62 patients with Ipc-PH or No-PH patients. Demographic, diagnostic, and etiological comparisons between the two groups were consistent with those observed based on the 7th WSPH criteria, with no significant differences noted. However, Ipc-PH patients exhibited a higher prevalence of VHD. The hemodynamic differences were also aligned with those observed based on the 7th WSPH criteria (Table 1).

### Characteristics of the derivation cohort

In accordance with the 7th WSPH criteria, patients diagnosed with Cpc-PH demonstrated significantly elevated measurements for LAD, RVEDTD, mitral valve E/Em ratio, LVFS, PASP, and TR Vmax. Notably, although the mean values for RALD, IVS and RVFWT were comparable between the two groups, minor discrepancies persisted (Table 2).

**TABLE 2:** Echocardiographic parameters of patients: a comparison between the 7th World Symposium on Pulmonary Hypertension (WSPH) criteria and the 2015 European Society of Cardiology/European Respiratory Society (ESC/ERS) criteria.

Variable	2015 ESC/ERS criteria			7th WSPH criteria		
	Ipc-PH/No-PH	Cpc-PH	Cut-off	Ipc-PH/No-PH	Cpc-PH	Cut-off
	(*n* = 136)	(*n* = 62)		(*n* = 93)	(*n* = 105)	
LAD, cm	4.3 ± 0.7	4.4 ± 0.8	5.0	4.2 ± 0.7	4.4 ± 0.8	**5.3** ^***^
LVEDD, cm	5.0 ± 0.8	4.8 ± 1.0	6.7	5.0 ± 0.9	5.0 ± 0.9	5.3
LVESD, cm	3.2 ± 0.8	3.0 ± 0.9	3.3	3.1 ± 0.8	3.2 ± 0.9	3.3
LVPWT, cm	0.9 (0.7, 1.0)	0.8 (0.7, 1.0)	1.4	0.9 (0.7, 1.0)	0.8 (0.7, 1.0)	1.2
RAP, mmHg	8.0 (5.0, 8.0)	8.0 (5.0, 9.5)	8.1	8.0 (5.0, 8.0)	8.0 (5.0, 8.1)	8.1
RATD, cm	4.3 (3.8, 5.0)	4.4 (4.0, 5.0)	**4.1** ^**^	4.4 (3.8, 5.3)	4.3 (3.9, 4.9)	3.9
RALD, cm	5.4 (4.7, 6.2)	5.4 (4.8, 6.1)	5.2	5.4 (4.6, 6.4)	5.4 (4.9, 6.1)	**4.9** ^**^
RVEDTD, cm	3.6 (3.1, 4.0)	3.7 (3.4, 4.2)	**3.6** ^**^	3.5 (3.1, 4.0)	3.7 (3.2, 4.0)	**3.6** *
RVEDLD, cm	6.3 (5.7, 7.1)	6.6 (5.7, 7.0)	6.4	6.3 (5.7, 7.1)	6.5 (5.7, 7.0)	7.5
IVS, cm	0.8 (0.7, 1.0)	0.8 (0.7, 1.0)	1.2	0.8 (0.7, 1.0)	0.8 (0.7, 1.0)	**1.2** ^**^
RVFWT, mm	0.6 (0.5, 0.7)	0.6 (0.5, 0.7)	0.9	0.6 (0.5, 0.7)	0.6 (0.5, 0.7)	**0.7** ^**^
TAPSE, cm	1.9 ± 0.5	1.8 ± 0.3	1.6	1.9 ± 0.5	1.8 ± 0.4	1.4
MV Sm, cm/s	9.0 (7.0, 10.0)	7.5 (7.0, 9.0)	15.0	9.0 (7.0, 11.0)	8.0 (7.0, 9.0)	5.0
MV Em, cm/s	10.0 (8.0, 12.0)	8.0 (6.2, 10.0)	3.0	10.0 (8.0, 12.0)	9.0 (7.0, 11.0)	5.0
MV E/Em	10.6 (8.0, 13.1)	12.2 (9.3, 15.5)	**12.2** ^***^	9.9 (7.6, 12.1)	12.1 (9.0, 15.8)	**12.6** ^***^
TV Sm, cm/s	11.0 (9.2, 13.0)	10.0 (9.0, 12.0)	7.0	11.0 (9.0, 13.0)	11.0 (9.0, 12.0)	7.0
ENDSEI	1.0 (1.0, 1.1)	1.0 (1.0, 1.1)	1.05	1.0 (1.0, 1.1)	1.0 (1.0, 1.1)	1.09
LVEF, %	66.1 ± 11.7	66.3 ± 12.0	77.0	66.6 ± 11.6	65.8 ± 12.0	58.0
LVFS, %	40.0 (33.0, 47.0)	41.0 (33.2, 47.8)	32.0	40.0 (32.0, 47.0)	41.0 (34.0, 48.0)	**30.0** ^**^
AA, cm	2.7 ± 0.5	2.6 ± 0.4	2.6	2.7 ± 0.4	2.7 ± 0.5	3.3
PA, cm	2.8 (2.0, 3.0)	2.8 (2.5, 3.1)	3.0	2.8 (2.5, 3.2)	2.8 (2.4, 3.1)	3.0
PASP, mmHg	50.0 (41.0, 57.0)	58.5 (47.2, 76.0)	**57.0** ^***^	49.0 (40.0, 56.0)	56.0 (45.0, 70.0)	**57.0** ^***^
TR Vmax, cm/s	325.0 (289.5, 350.5)	363.0 (322.5, 405.0)	**355.0** ^***^	315.0 (283.0, 347.0)	342.0 (311.0, 394.0)	**352.0** ^***^
TAPSE:PASP, mm/mmHg	0.04 (0.03, 0.05)	0.03 (0.02, 0.04)	**0.014** ^**^	0.04 (0.03, 0.05)	0.03 (0.03, 0.04)	**0.017** ^**^

Values are expressed as the mean ± SD or median (Interquartile range). AO, aortic diameter; ENDSEI, end-diastolic stage eccentricity index; IVS, interventricular septum; LAESD, left atrial end-systolic diameter; LVEDD, left ventricular end-diastolic diameter; LVEF, left ventricular ejection fraction; LVFS, left ventricular fractional shortening; LVPWT, left ventricular posterior wall thickness; MV Em, mitral valve early diastolic wave velocity; MV E/Em, mitral valve early diastolic to early diastolic annular velocity ratio; MV Sm, mitral valve systolic wave velocity; PA, pulmonary artery diameter; PASP, pulmonary artery systolic pressure; RATD, right atrial transverse diameter; RALD, right atrial longitudinal diameter; RAP, right atrial pressure; RVEDLD, right ventricular end-diastolic longitudinal dimension; RVEDTD, right ventricular end-diastolic transverse dimension; RVFWT, right ventricular free wall thickness; TAPSE, tricuspid annular plane systolic excursion; TR Vmax, tricuspid regurgitation peak velocity; TV Sm, tricuspid valve systolic wave velocity.

*P < 0.1,

**P < 0.05,

***P < 0.01.

Per the 2015 ESC/ERS criteria, the differences in echocardiographic parameters between patients with Cpc-PH and Ipc-PH were less marked compared to those noted within the 7th WSPH criteria cohort. Specifically, Cpc-PH patients exhibited higher values for right RATD, RVEDTD, mitral valve E/Em ratio, PASP, and TR Vmax (Table 2).

### Development of the PECS model

In accordance with the 7th WSPH criteria, within the cohort analysis of the 7th WSPH group, although bronchial asthma, RALD, and RVEDTD achieved statistical significance or marginal significance in univariate analysis (*p*-values of 0.068, 0.051, and 0.059, respectively), they failed to meet the L2 regularization and stepwise backward logistic regression screening and were therefore excluded. A total of six variables were identified as statistically significant in multivariate analysis: VHD (*P* = 0.012), LAD (*P* = 0.057), IVS (*P* = 0.026), mitral valve E/Em ratio (*P* = 0.026), LVFS (*P* = 0.015), and TR Vmax (*P* < 0.001). The PECS was derived from the regression coefficients of these variables in the final model, with the following weighted scores: PECS_7th WSPH_ = 0.9 × PECS_VHD_ + 1.2 × PECS_LAD_ + 1.6 × PECS_IVS_ + 0.8 × PECS_MV E/Em_ + 1.2 × PECS_LVFS_ + 1.4 × PECS_TR Vmax_ (Table 3).

**TABLE 3:** Logistic regression analysis of echocardiographic parameters according to the 7th World Symposium on Pulmonary Hypertension (WSPH) criteria.

Variable	Univariate analysis			Multivariate analysis			
	β-coefficient	*P*-value	OR (95% CI)	β-coefficient	*P*-value	OR (95% CI)	Weighted scores
VHD	0.799	0.007	2.22 (1.25, 3.97)	0.858	0.012	2.36 (1.21, 4.60)	0.9
BA	−1.112	0.068	0.33 (0.10, 1.09)				
MR	1.146	0.052	3.14 (0.99, 10.01)				
LAD, cm	1.458	0.011	4.30 (1.39, 13.28)	1.192	0.057	3.29 [0.97, 11.21]	1.2
RALD, cm	0.635	0.051	1.89 (1.00, 3.57)				
RVEDTD, cm	0.548	0.059	1.73 (0.98, 3.05)				
IVS, cm	1.256	0.06	3.51 (0.95, 13.00)	1.634	0.026	5.12 (1.21, 21.66)	1.6
RVFWT, mm	0.752	0.014	2.12 (1.17, 3.86)				
MV E/Em	1.022	0.001	2.78 (1.49, 5.16)	0.811	0.026	2.25 (1.10, 4.59)	0.8
LVFS, %	1.008	0.02	2.74 (1.17, 6.40)	1.22	0.015	3.39 (1.27, 9.02)	1.2
PASP, mmHg	1.153	< 0.001	3.17 (1.72, 5.84)				
TR Vmax, cm/s	1.37	< 0.001	3.94 (2.07, 7.47)	1.377	< 0.001	3.96 (1.95, 8.07)	1.4

Variables were dichotomized according to Youden index-derived optimal cut-off values. If VHD = 1, weighted score = +0.9; if LAD ≥ 5.3 cm, weighted score = +1.2; if IVS ≥ 1.2 cm, weighted score = +1.6; if MV E/Em ≥ 12.6, weighted score = +0.8; if LVFS ≥ 30%, weighted score = +1.2; if TR Vmax ≥ 352 cm/s, weighted score = +1.4. Abbreviations as in Tables 1, 2.

In the derivation cohort of the 2015 ESC/ERS criteria, multivariate logistic regression revealed four significant variables: VHD (*P* = 0.095), RVEDTD (*P* = 0.087), mitral valve E/Em ratio (*P* = 0.037), and TR Vmax (*P* < 0.001). The PECS_2015ESC/ERS_ model formula was established based on their β-coefficient values: PECS_2015ESC/ERS_ = 0.6 × PECS_VHD_ + 0.6 × PECS_RVEDTD_ + 0.7 × PECS_MV E/Em_ + 1.3 × PECS_TR Vmax_ (Table 4).

**TABLE 4:** Logistic regression analysis of echocardiographic Parameters based on the 2015 European Society of Cardiology/European Respiratory Society (ESC/ERS) criteria.

Variable	Univariate analysis			Multivariate analysis			
	β-coefficient	*P*-value	OR [95% CI]	β-coefficient	*P*-value	OR [95% CI]	Weighted scores
Valvular heart disease	0.544	0.079	1.72 (0.94, 3.16)	0.568	0.095	1.76 (0.91, 3.44)	0.6
RATD, cm	0.669	0.048	1.95 (1.00, 3.79)				
RVEDTD, cm	0.787	0.015	2.20 (1.16, 4.15)	0.602	0.087	1.83 (0.92, 3.64)	0.6
MV E/Em	0.911	0.004	2.49 (1.34, 4.63)	0.713	0.037	2.04 (1.04, 4.00)	0.7
PASP, mmHg	1.376	< 0.001	3.96 (2.10, 7.45)				
TR Vmax, cm/s	1.414	< 0.001	4.11 (2.17, 7.81)	1.275	< 0.001	3.58 (1.82, 7.04)	1.3

Variables were dichotomized according to Youden index-derived optimal cut-off values. If VHD = 1, weighted score = +0.6; if RVEDTD ≥ 3.6 cm, weighted score = +0.6; if MV E/Em ≥ 12.2, weighted score = +0.7; if TR Vmax ≥ 355 cm/s, weighted score = +1.3. Abbreviations as in Tables 1, 2.

### Evaluation of the PECS model

The PECS_7th WSPH_ model was evaluated using ROC curve analysis. At a cutoff score of ≥ 2.2, the model exhibited diagnostic efficacy characterized by a sensitivity of 66.7%, specificity of 72.0%, PPV of 72.9%, NPV of 65.7%, accuracy of 69.2%, and an AUC of 0.761 (95% CI: 0.692–0.823, *P* < 0.001) (Supplementary Table 1).

The PECS_2015ESC/ERS_ model was assessed using ROC curve analysis. With a cutoff score of ≥ 1.3, the model exhibited sensitivity of 64.50%, specificity of 72.8%, PPV of 51.9%, NPV of 81.8%, accuracy of 70.2%, and an AUC of 0.733 (95% CI: 0.657–0.808, *P* < 0.001) (Supplementary Table 2).

**FIGURE 2:**
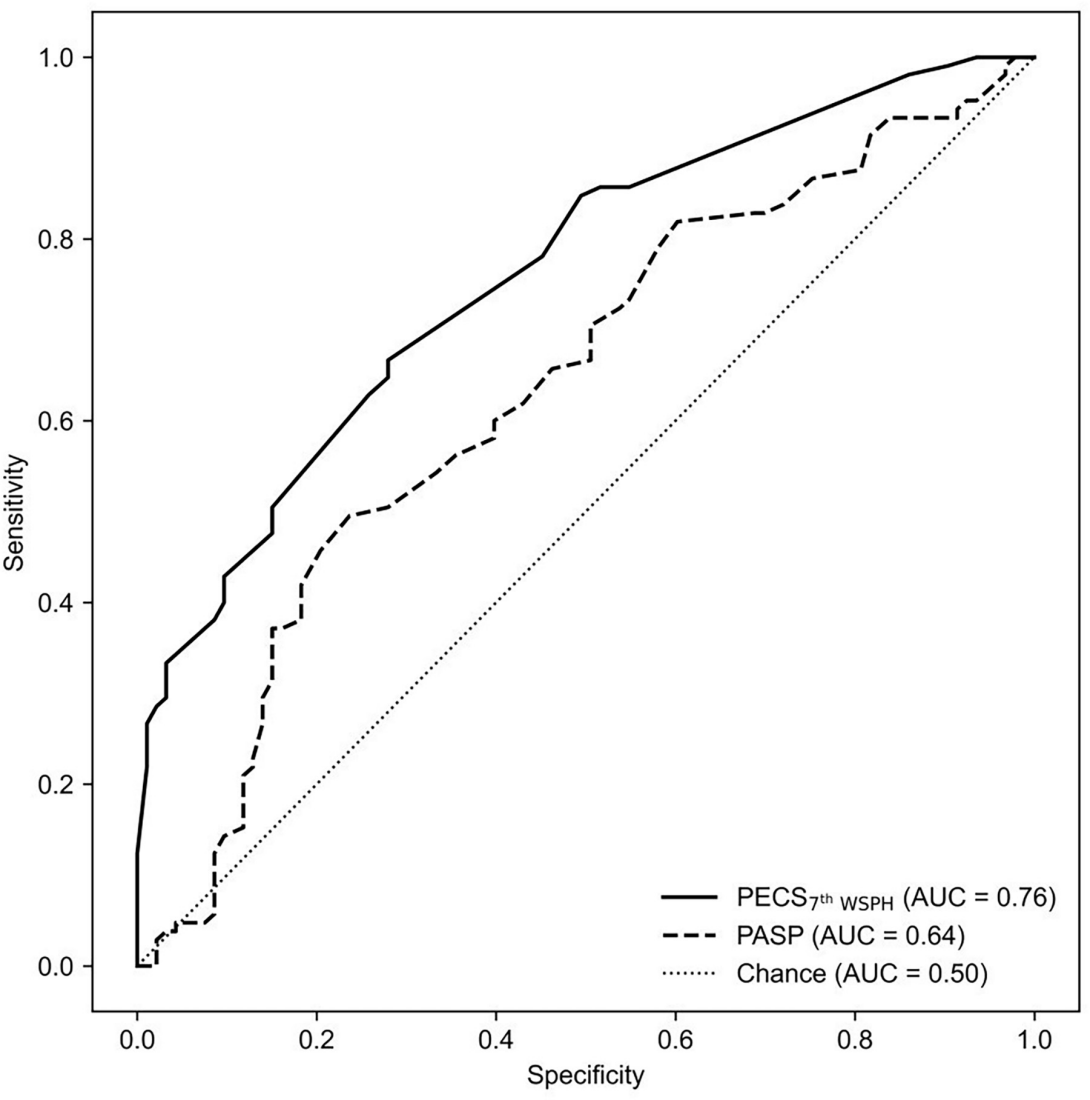
The ROC curve is shown for PECS_7th WSPH_ and for PASP alone as determined by echocardiography variables in predicting Cpc-PH. AUC, area under the curve; PECS, Predictive Echocardiography Cpc-PH Score; PASP, pulmonary arterial systolic pressure; Cpc-PH, combined post-capillary pulmonary hypertension; ROC, receiver-operator characteristic.

**FIGURE 3:**
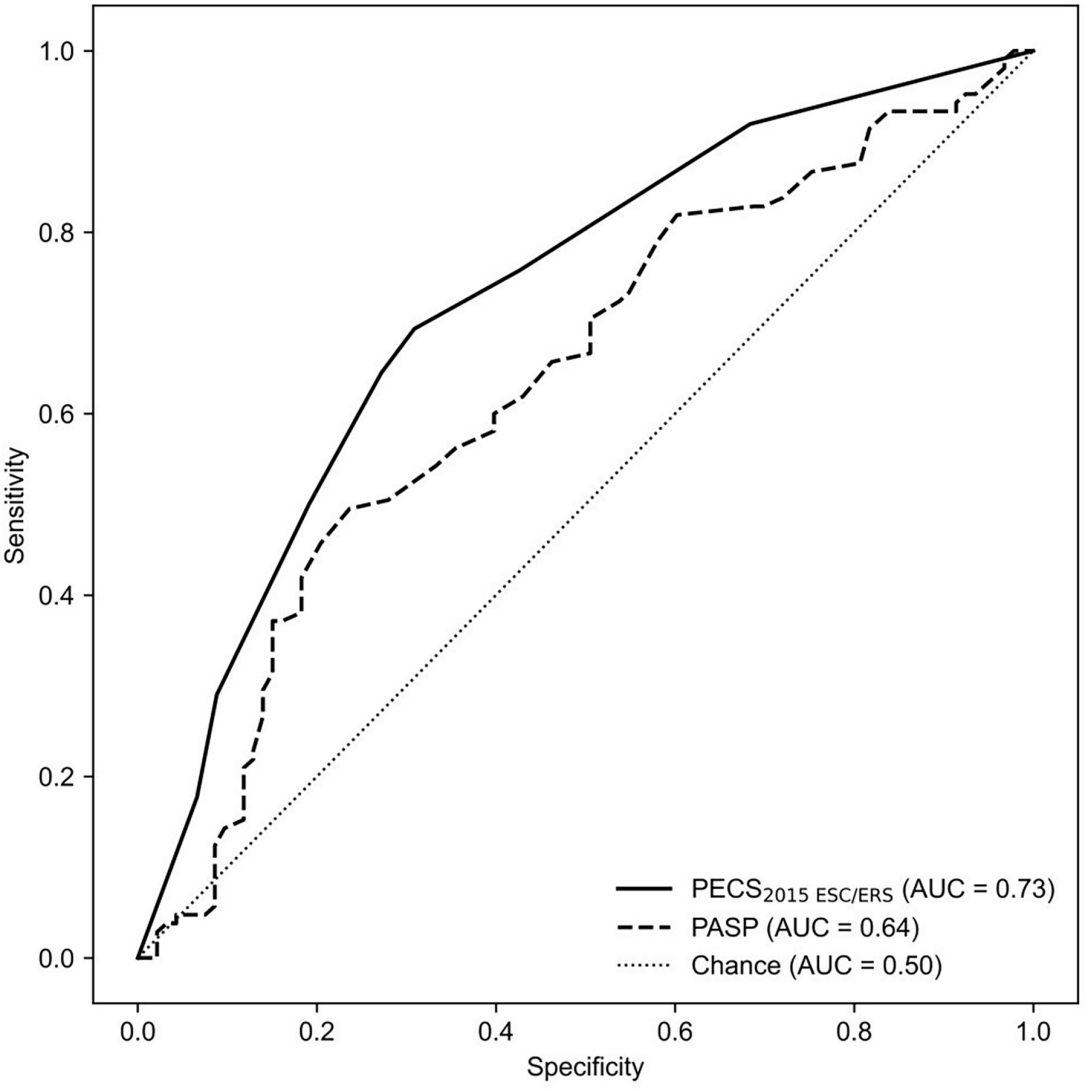
The ROC curve is shown for PECS_2015ESC/ERS_ and for PASP alone as determined by echocardiography variables in predicting Cpc-PH. AUC, area under the curve; PECS, Predictive Echocardiography Cpc-PH Score; PASP, pulmonary arterial systolic pressure; Cpc-PH, combined post-capillary pulmonary hypertension; ROC, receiver-operator characteristic.

Under the 2015 ESC/ERS criteria, the AUC of PASP was 0.662 (95% CI: 0.494–0.65), while the model achieved an AUC of 0.733 (95% CI: 0.657–0.808, *P* < 0.001), marking an AUC improvement of 10.73% (95% CI: 1.47%–21.27%, *P* < 0.001) (Figure 2). Under the 7th WSPH criteria, the AUC of PASP was 0.629 (95% CI: 0.566–0.695), while the model achieved an AUC of 0.761 (95% CI: 0.692–0.823, *P* < 0.001), with an AUC improvement of 20.99% (95% CI: 9.54%–33.73%, *P* < 0.001) (Figure 3). However, the Z-test showed no significant difference between the models under the two criteria (*P* = 0.521) (Supplementary Figure 1).

### 5-fold cross-validation analysis

Following the constructing prediction models based on both the 2015 ESC/ERS and the 7th WSPH criteria, a 5-fold cross-validation approach was utilized to assess the efficacy of the two models. The performance metrics for the PECS_7th WSPH_ model, were as follows: AUC, 0.752 ± 0.070; sensitivity, 0.657 ± 0.097; specificity, 0.653 ± 0.170; PPV, 0.693 ± 0.089; NPV, 0.623 ± 0.078; accuracy, 0.656 ± 0.081 (Supplementary Table 3).

For the PECS_2015ESC/ERS_ model, the performance metrics from 5-fold cross-validation were as follows: AUC, 0.694 ± 0.082; sensitivity, 0.276 ± 0.086; specificity, 0.883 ± 0.084; PPV, 0.575 ± 0.284; NPV, 0.727 ± 0.039; accuracy, 0.692 ± 0.076 (Supplementary Table 4).

### Nomograms

**FIGURE 4:**
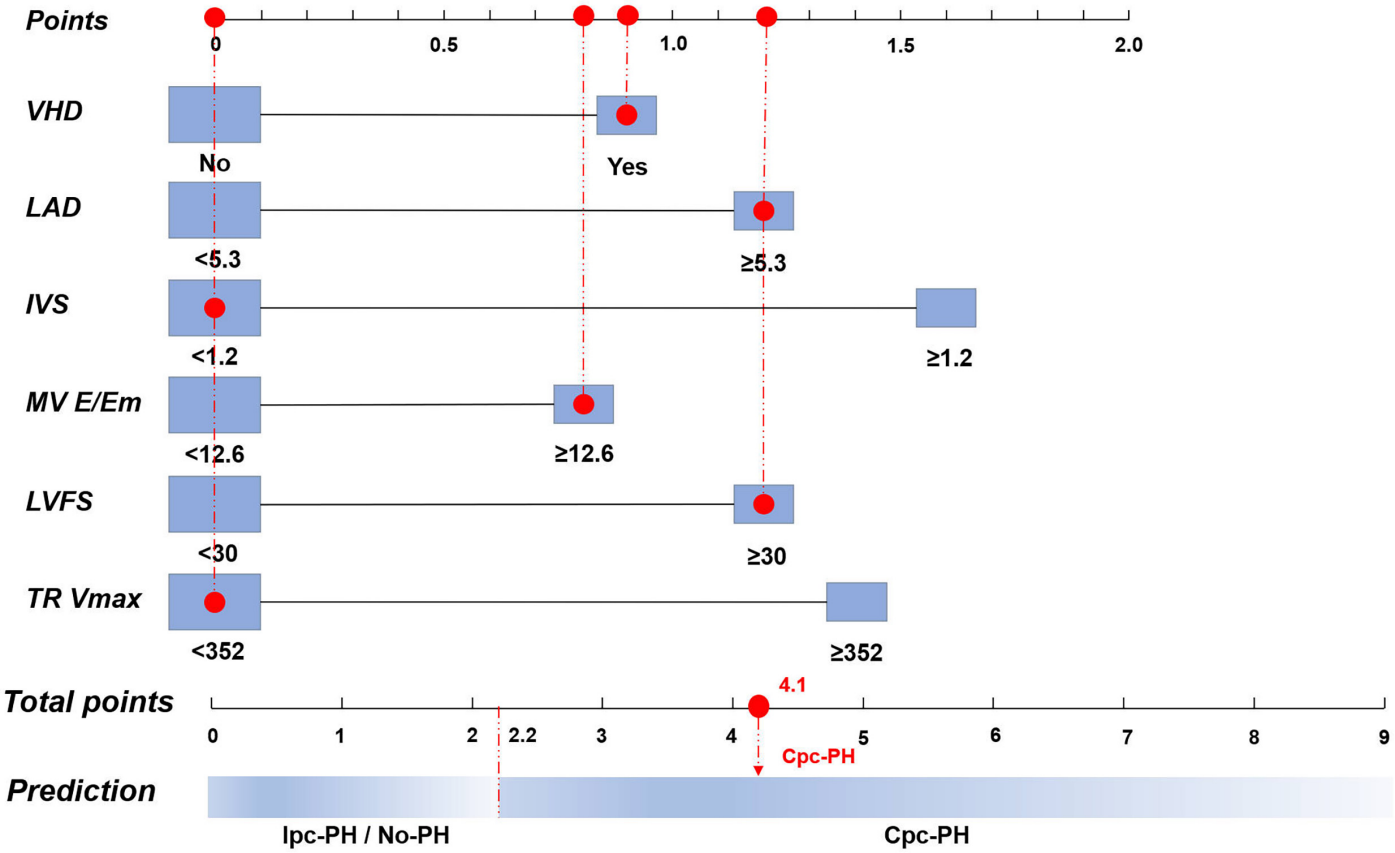
Nomogram of the logistic regression model based on the 7th World Symposium on Pulmonary Hypertension (WSPH) criteria. VHD, valvular heart disease; LAD, left atrial diameter; IVS, interventricular septum; MV E/Em, mitral valve early diastolic to early diastolic annular velocity ratio; LVFS, left ventricular fractional shortening; TR Vmax, tricuspid regurgitation peak velocity; Cpc-PH, combined post-capillary pulmonary hypertension; Ipc-PH, isolated post-capillary pulmonary hypertension.

**FIGURE 5:**
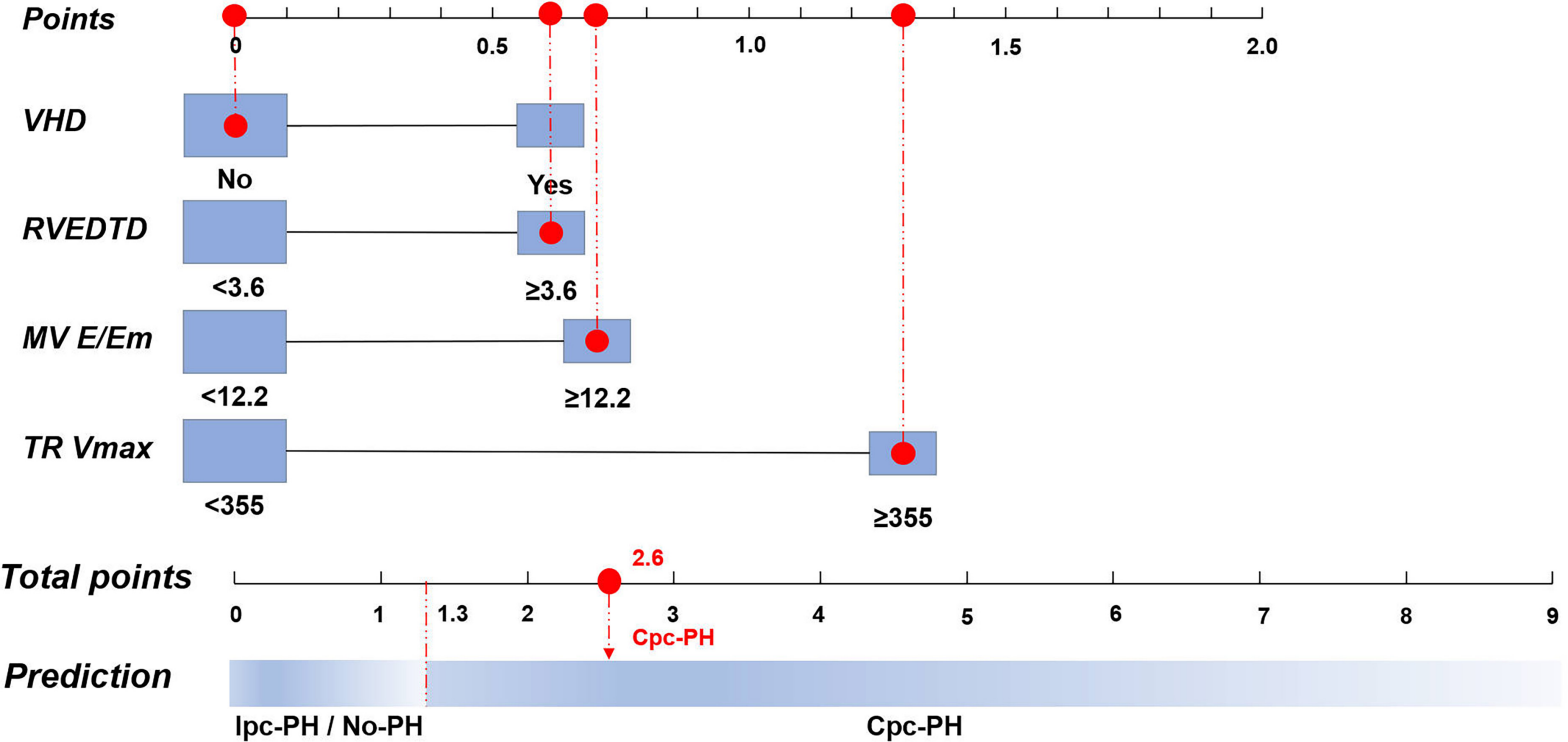
Nomogram of the logistic regression model based on the 2015 European Society of Cardiology/European Respiratory Society (ESC/ERS) Criteria. VHD, valvular heart disease; RVEDTD, right ventricular end-diastolic transverse dimension; MV E/Em, mitral valve early diastolic to early diastolic annular velocity ratio; TR Vmax, tricuspid regurgitation peak velocity; Cpc-PH, combined post-capillary pulmonary hypertension; Ipc-PH, isolated post-capillary pulmonary hypertension.

To facilitate clinical decision-making, nomograms were developed based on both the 7th WSPH and the 2015 ESC/ERS criteria. The 7th WSPH-based nomogram includes variables such as VHD, LAD, IVS, MV E/Em ratio, LVFS, and TR Vmax. PECS_7th WSPH_ ≥ 2.2 strongly predicts the presence of Cpc-PH (Figure 4). The nomogram based on the 2015 ESC/ERS criteria uses variables including VHD, RVEDTD, MV E/Em ratio, and TR Vmax. PECS_2015ESC/ERS_ ≥ 1.3 predicts Cpc-PH (Figure 5).

## Discussion

In this study, the PECS model was developed and validated against both the 7th WSPH and the 2015 ESC/ERS criteria. It proved more effective than PASP for detecting Cpc-PH. Facilitating clinical decisions with a risk score indicates substantial innovation and practical value. However, there was no significant difference between the diagnostic performance of the PECS_7th WSPH_ and the PECS_2015ESC/ERS_ models.

In this study, we did not separately analyze no-PH and lpc-PH patients. The primary reason is that Cpc-PH, as a severe form of PH caused by left heart disease, has different treatment strategies compared to lpc-PH and no-PH (1, 7, 25).

The 2015 ESC/ERS criteria for Cpc-PH included PAWP > 15 mmHg, mPAP ≥ 25 mmHg, and PVR ≥ 3 WU, while the 7th WSPH and 2022 ESC/ERS criteria reduced these to PAWP > 15 mmHg, mPAP > 20 mmHg, and PVR > 2 Wood units. This shift in thresholds significantly affected the diagnostic landscape for Cpc-PH patients. Studies have indicated that reducing the PVR threshold from 3 to 2 WU could increase the identification rate of Cpc-PH patients by approximately 60% (26). In this study, we developed the PECS models based on the 7th WSPH and 2015 ESC/ERS criteria. Findings revealed that both models exhibited similar discriminative accuracy, with AUC values of 0.761 and 0.727, respectively, and the difference between them was not statistically significant. However, discrepancies were observed in the balance between sensitivity and specificity for the models. The PECS model adopting the updated criteria placed greater emphasis on enhancing sensitivity, achieving an average sensitivity of approximately 66% in 5-fold cross-validation, markedly higher than the 28% of the previous criteria model; correspondingly, the specificity of the new model was around 65%, slightly lower than that of the old model at 88%. This suggests that as diagnostic thresholds are lowered, the model must recognize a greater number of patients with relatively milder Cpc-PH, thereby enhancing the detection rate of potential cases, but also resulting in an increase in false positives. Under the previous criteria, the model was more stringent, primarily identifying typical Cpc-PH cases with higher PVR, hence exhibiting higher specificity but also a greater risk of failing to diagnose. Furthermore, the PECS_7th WSPH_ model demonstrated greater stability in cross-validation, indicating that the model’s applicability across various population was instead strengthened.

The PECS model in this study provides innovative and superior non-invasive predictive capabilities compared to prior methods. PECS_7th WSPH_ achieved an AUC of 0.761, clearly exceeding routine PASP estimation (AUC: 0.629) and performing competitively against representative non-invasive indices (Supplementary Table 5) (27, 28). In this study, we utilized regularized logistic regression to identify six salient parameters: VHD, LAD, IVS, mitral valve E/Em ratio, LVFS, and TV max. This approach culminated in the development of the novel PECS_7th WSPH_ scoring system. Furthermore, given its inclusion of medical history and multi-scale echocardiographic parameters, the PECS model provides a more reliable assessment in the screening of Cpc-PH, demonstrating a certain degree of innovation and practical value.

Clinically, the PECS _7th WSPH_ model offers a promising avenue for early detection and intervention of Cpc-PH. Although RHC remains the gold standard for diagnosing PH, it is an invasive procedure associated with potential complications (29). Elderly, frail patients or those with multiple comorbidities often cannot tolerate RHC, which can result in delayed diagnosis and consequently, delayed treatment of manageable PH (29). In contrast, echocardiography is a non-invasive, safe, and widely accessible diagnostic modality that serves as an essential tool for the screening of PH (10). The PECS model, based on conventional ultrasound parameters, offers a practical solution for identifying Cpc-PH. Scores are easily calculated through routine ultrasound assessments and medical history collection. For patients suspected of Group 2 PH, this model can guide decisions on whether to proceed with invasive diagnostic procedures. A high PECS score supports the need for RHC to confirm diagnosis and initiate intervention, while a low score allows for close monitoring without unnecessary invasive tests. The PECS model is poised to enhance prognostic evaluation and guide therapy for pcPH patients, particularly those at risk of Cpc-PH. It underscores the need for rigorous management of underlying conditions and close monitoring of pulmonary vascular factors. The ultrasound-based PECS model emerges as a valuable non-invasive screening tool, boosting detection rates in high-risk groups and holding significant promise for clinical application.

A multicenter prospective study has confirmed that this classification method holds considerable prognostic value for the outcomes of patients with pcPH (26).

This study is subject to several limitations that delineate directions for future research. Firstly, the retrospective, single-center nature of the study results in a limited sample size, and the sensitivity/specificity of the model is modest. The RHC is an invasive diagnostic procedure, which poses practical challenges, especially when performed in elderly patients with multiple comorbidities. These challenges directly restrict the number of patients that can be included in our study. Despite these constraints, we have included all patients who met the criteria for this study, totaling 198 cases, of which 105 were confirmed as Cpc-PH. Our model incorporates six predictive factors, and the ratio of events to variables is approximately 17.5, which exceeds the generally accepted minimum threshold of 10. From a statistical perspective, we deem the current sample size to be adequate for model construction. Secondly, the model’s predictive factors are predominantly derived from echocardiographic and clinical history data, excluding serum biomarkers (e.g., BNP, NT-proBNP) and cardiopulmonary exercise test results. This exclusion may limit the model’s comprehensive assessment of Cpc-PH. To fully establish the model’s clinical utility, external validation in multicenter cohorts with diverse patient demographics is essential. Thirdly, in our study, there is a lack of external validation. Ongoing prospective validation and refinement of the model are anticipated to enhance its robustness and accuracy, ultimately positioning it as a valuable non-invasive screening tool for Cpc-PH. We have employed five-fold cross-validation as an internal validation method to assess the model’s stability. The cross-validation yielded an average AUC of approximately 0.75, which is very close to the model’s AUC on the entire derivation cohort (0.761), indicating that the model has good stability. This internal validation has strengthened our confidence in the model’s reliability and, to some extent, compensates for the current lack of external validation.

## Conclusion

The study results indicate that the PECS is a valid and effective tool for identifying Cpc-PH, and it performs better than PASP. The PECS can be useful for clinicians in the diagnosis and management of patients with Cpc-PH.

## Data Availability

The raw data supporting the conclusions of this article will be made available by the authors, without undue reservation.

## Supplementary material

Supplementary Figure 1 The ROC curve is shown for PECS7th WSPH and for PECS2015 ESC/ERS in predicting Cpc-PH. AUC: area under the curve; PECS: Predictive Echocardiography Cpc-PH Score; Cpc-PH, combined post-capillary pulmonary hypertension; ROC: receiver-operator characteristic.
