# Supplementary figures and images for: Non-invasive PECS model for detection of combined post-capillary pulmonary hypertension
Source: Front Med (Lausanne). 2025 Oct 22;12:1660387. doi: 10.3389/fmed.2025.1660387 (PMC12585943; doi:10.3389/fmed.2025.1660387)

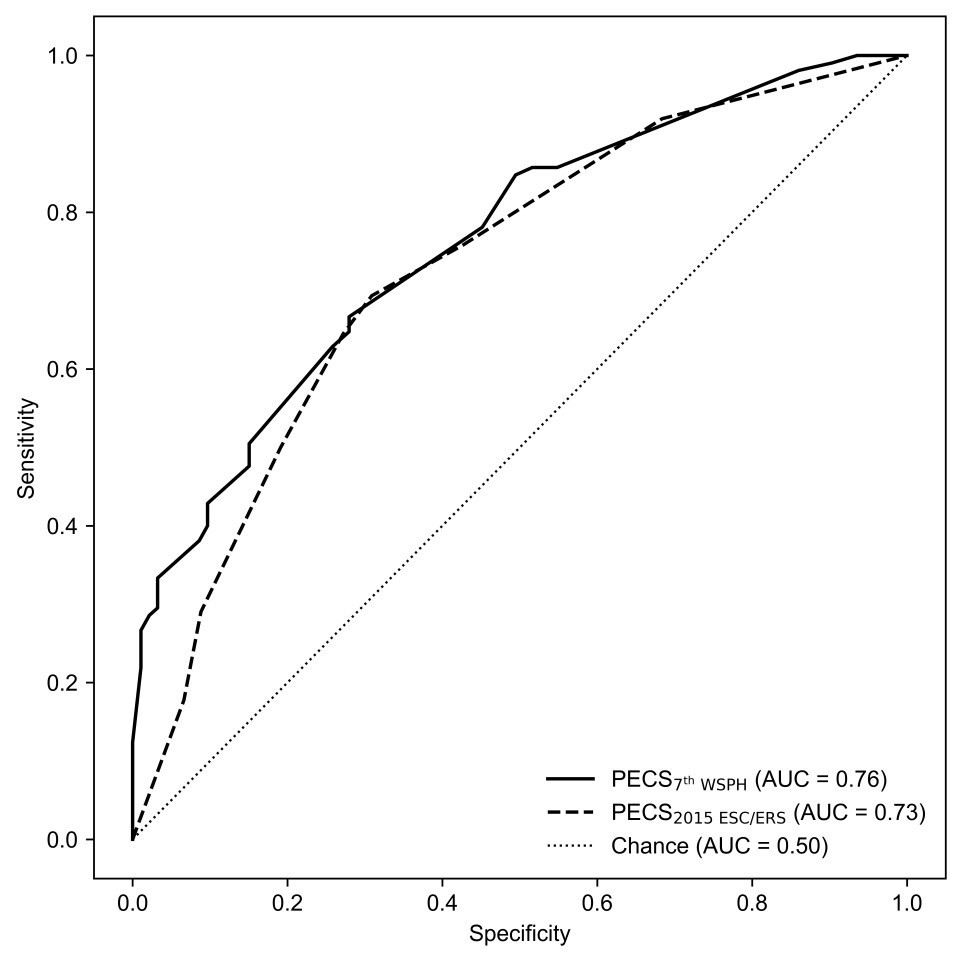

Supplement: Supplementary Figure 1 — The ROC curve is shown for PECS7th WSPH and for PECS2015 ESC/ERS in predicting Cpc-PH. AUC: area under the curve; PECS: Predictive Echocardiography Cpc-PH Score; Cpc-PH, combined post-capillary pulmonary hypertension; ROC: receiver-operator characteristic. [file Image_1.jpeg]
