# Supplementary material for: Non-invasive PECS model for detection of combined post-capillary pulmonary hypertension
Source: Front Med (Lausanne). 2025 Oct 22;12:1660387. doi: 10.3389/fmed.2025.1660387 (PMC12585943; doi:10.3389/fmed.2025.1660387)
Supplement: Supplementary file 2 [file Table_1.docx]

| Supplementary Table 1. Echocardiographic Parameter Distribution and Discriminative Accuracy based on the 7th WSPH Criteria | | | | | | | | | | |
| --- | --- | --- | --- | --- | --- | --- | --- | --- | --- | --- |
| Variable | All Subjects | Ipc-PH/  No-PH | Cpc-PH | *P* | Sensitivity | Specificity | PPV | NPV | Accuracy | AUC |
|  | (n = 198) | (n = 93) | (n = 105) |  |  |  |  |  |  |  |
| PECS scores | | | | | | | | | | |
| < 2.2 | 102 (51.5) | 67 (72.0) | 35 (33.3) | < 0.001 | 0.667 | 0.72 | 0.729 | 0.657 | 0.692 | 0.761 [0.692, 0.823] |
| ≥ 2.2 | 96 (48.5) | 26 (28.0) | 70 (66.7) |  |  |  |  |  |  |  |
| VHD | | | | | | | | | | |
| 0 | 114 (57.6) | 63 (67.7) | 51 (48.6) | 0.007 | 0.514 | 0.677 | 0.643 | 0.553 | 0.591 | 0.596 [0.527, 0.667] |
| 1 | 84 (42.4) | 30 (32.3) | 54 (51.4) |  |  |  |  |  |  |  |
| LAD, cm | | | | | | | | | | |
| < 5.3 | 177 (89.4) | 89 (95.7) | 88 (83.8) | 0.011 | 0.162 | 0.957 | 0.81 | 0.503 | 0.535 | 0.559 [0.52, 0.6] |
| ≥ 5.3 | 21 (10.6) | 4 (4.3) | 17 (16.2) |  |  |  |  |  |  |  |
| IVS,cm | | | | | | | | | | |
| < 1.2 | 184 (92.9) | 90 (96.8) | 94 (89.5) | 0.060 | 0.105 | 0.968 | 0.786 | 0.489 | 0.51 | 0.536 [0.499, 0.572] |
| ≥ 1.2 | 14 (7.1) | 3 (3.2) | 11 (10.5) |  |  |  |  |  |  |  |
| MV E/Em | | | | | | | | | | |
| < 12.6 | 130 (65.7) | 72 (77.4) | 58 (55.2) | 0.001 | 0.448 | 0.774 | 0.691 | 0.554 | 0.601 | 0.611 [0.547, 0.671] |
| ≥ 12.6 | 68 (34.3) | 21 (22.6) | 47 (44.8) |  |  |  |  |  |  |  |
| LVFS,% | | | | | | | | | | |
| < 30 | 28 (14.1) | 19 (20.4) | 9 (8.6) | 0.02 | 0.914 | 0.204 | 0.565 | 0.679 | 0.581 | 0.559 [0.513, 0.607] |
| ≥ 30 | 170 (85.9) | 74 (79.6) | 96 (91.4) |  |  |  |  |  |  |  |
| TR Vmax, cm/s | | | | | | | | | | |
| < 352 | 129 (65.2) | 75 (80.7) | 54 (51.4) | < 0.001 | 0.486 | 0.806 | 0.739 | 0.581 | 0.636 | 0.646 [0.587, 0.712] |
| ≥ 352 | 69 (34.9) | 18 (19.4) | 51 (48.6) |  |  |  |  |  |  |  |
| Data in parentheses are 95% confidence intervals (CIs). Abbreviations as in Table 1 and 2. AUC, area under the curve; PPV: positive predictive value; NPV: negative predictive value. | | | | | | | | | | |
