# Supplementary material for: Non-invasive PECS model for detection of combined post-capillary pulmonary hypertension
Source: Front Med (Lausanne). 2025 Oct 22;12:1660387. doi: 10.3389/fmed.2025.1660387 (PMC12585943; doi:10.3389/fmed.2025.1660387)
Supplement: Supplementary file 3 [file Table_2.docx]

| Supplementary Table 2 Echocardiographic Parameter Distribution and Discriminative Accuracy based on the 2015 ESC/ERS Criteria | | | | | | | | | | |
| --- | --- | --- | --- | --- | --- | --- | --- | --- | --- | --- |
| Variable | All Subjects | Ipc-PH/  No-PH | Cpc-PH | P | Sensitivity | Specificity | PPV | NPV | Accuracy | AUC |
|  | (n = 198) | (n = 136) | (n = 62) |  |  |  |  |  |  |  |
| PECS scores | | | | | | | | | | |
| < 1.3 | 121 (61.1) | 99 (72.8) | 22 (35.5) | < 0.001 | 0.645 | 0.728 | 0.519 | 0.818 | 0.702 | 0.733 [0.657, 0.808] |
| ≥ 1.3 | 77 (38.9) | 37 (27.2) | 40 (64.5) |  |  |  |  |  |  |  |
| VHD | | | | | | | | | | |
| 0 | 114 (57.6) | 84 (61.8) | 30 (48.4) | 0.079 | 0.516 | 0.618 | 0.381 | 0.737 | 0.586 | 0.567 [0.494, 0.65] |
| 1 | 84 (42.4) | 52 (38.2) | 32 (51.6) |  |  |  |  |  |  |  |
| RVEDTD, cm | | | | | | | | | | |
| < 3.6 | 86 (43.4) | 67 (49.3) | 19 (30.7) | 0.015 | 0.694 | 0.493 | 0.384 | 0.779 | 0.556 | 0.593 [0.52, 0.666] |
| ≥ 3.6 | 112 (56.6) | 69 (50.7) | 43 (69.4) |  |  |  |  |  |  |  |
| MV E/Em | | | | | | | | | | |
| < 12.2 | 128 (64.7) | 97 (71.3) | 31 (50.0) | 0.004 | 0.5 | 0.713 | 0.443 | 0.758 | 0.646 | 0.607 [0.529, 0.678] |
| ≥ 12.2 | 70 (35.4) | 39 (28.7) | 31 (50.0) |  |  |  |  |  |  |  |
| TR Vmax, cm/s | | | | | | | | | | |
| < 355 | 133 (67.2) | 105 (77.2) | 28 (45.2) | < 0.001 | 0.548 | 0.772 | 0.523 | 0.789 | 0.702 | 0.66 [0.586, 0.734] |
| ≥ 355 | 65 (32.8) | 31 (22.8) | 34 (54.8) |  |  |  |  |  |  |  |
| Data in parentheses are 95% confidence intervals (CIs). Abbreviations as in Table 2. AUC, area under the curve; PPV: positive predictive value; NPV: negative predictive value. | | | | | | | | | | |
