# Supplementary material for: Non-invasive PECS model for detection of combined post-capillary pulmonary hypertension
Source: Front Med (Lausanne). 2025 Oct 22;12:1660387. doi: 10.3389/fmed.2025.1660387 (PMC12585943; doi:10.3389/fmed.2025.1660387)
Supplement: Supplementary file 4 [file Table_3.docx]

| Supplementary Table 3 Five-fold Cross-validation performance of the predictive model based on the 7th WSPH Criteria. | | | | | | |
| --- | --- | --- | --- | --- | --- | --- |
| FOLD | AUC | Sensitivity | Specificity | PPV | NPV | Accuracy |
| 1 | 0.744 | 0.524 | 0.684 | 0.647 | 0.565 | 0.6 |
| 2 | 0.883 | 0.762 | 0.842 | 0.842 | 0.762 | 0.8 |
| 3 | 0.753 | 0.667 | 0.684 | 0.7 | 0.65 | 0.675 |
| 4 | 0.693 | 0.571 | 0.722 | 0.706 | 0.591 | 0.641 |
| 5 | 0.689 | 0.762 | 0.333 | 0.571 | 0.545 | 0.564 |
| mean ± SD | 0.752 ± 0.070 | 0.657 ± 0.097 | 0.653 ± 0.170 | 0.693 ± 0.089 | 0.623 ± 0.078 | 0.656 ± 0.081 |
| AUC, area under the curve; PPV: positive predictive value; NPV: negative predictive value. | | | | | | |
