# Supplementary material for: Non-invasive PECS model for detection of combined post-capillary pulmonary hypertension
Source: Front Med (Lausanne). 2025 Oct 22;12:1660387. doi: 10.3389/fmed.2025.1660387 (PMC12585943; doi:10.3389/fmed.2025.1660387)
Supplement: Supplementary file 5 [file Table_4.docx]

| Supplementary Table 4 Five-fold Cross-validation performance of the predictive model based on the 2015 ESC/ERS Criteria. | | | | | | |
| --- | --- | --- | --- | --- | --- | --- |
| FOLD | AUC | Sensitivity | Specificity | PPV | NPV | Accuracy |
| 1 | 0.634 | 0.25 | 0.786 | 0.333 | 0.71 | 0.625 |
| 2 | 0.63 | 0.308 | 1 | 1 | 0.75 | 0.775 |
| 3 | 0.652 | 0.154 | 0.852 | 0.333 | 0.676 | 0.625 |
| 4 | 0.849 | 0.417 | 0.963 | 0.833 | 0.788 | 0.795 |
| 5 | 0.707 | 0.25 | 0.815 | 0.375 | 0.71 | 0.641 |
| mean ± SD | 0.694 ± 0.082 | 0.276 ± 0.086 | 0.883 ± 0.084 | 0.575 ± 0.284 | 0.727 ± 0.039 | 0.692 ± 0.076 |
| AUC, area under the curve; PPV: positive predictive value; NPV: negative predictive value. | | | | | | |
