# Supplementary material for: Non-invasive PECS model for detection of combined post-capillary pulmonary hypertension
Source: Front Med (Lausanne). 2025 Oct 22;12:1660387. doi: 10.3389/fmed.2025.1660387 (PMC12585943; doi:10.3389/fmed.2025.1660387)
Supplement: Supplementary file 6 [file Table_5.docx]

| Supplementary Table 5 Diagnostic Performance of different Non-Invasive Parameters and Models for Discriminating Types of PH. | | | | | | | |
| --- | --- | --- | --- | --- | --- | --- | --- |
| Author (Year) | Study design | Study population | Sample size by Cohort | | Guidelines | Best model / parameter | AUC |
|  |  |  | DC | VC |  |  |  |
| Kai'En Leong  (2022)^31^ | Retrospective cohort | Consecutive patients referred to a UK tertiary PH center (2015-2020). | 48 | NA | 2015 ESC/ERS | CMR LAVi | 0.96 (Pre- PH vs. Post-PH) |
| Csaba Jenei (2021)^32^ | Prospective cohort | Consecutive PH patients followed at a Hungarian tertiary cardiology center (2018-2020). | 75 | NA | 2015 ESC/ERS | LV EDVi+LAVi min | 0.89 (Pre-PH vs. Post-PH) |
| Ashwin Venkateshvaran (2019)^27^ | Prospective cohort | Consecutive patients referred at a Swedish tertiary university hospital (2014-2018). | 130 | NA | 2015 ESC/ERS | ePLAGS | 0.65 (Ipc-PH vs. Cpc-PH) |
| Gregory M Scalia (2016)^33^ | Retrospective cohort | Consecutive PH patients complete echocardiographic data. | 133 | NA | 2015 ESC/ERS | ePLAR | 0.87 (Pre-PH vs. Post-PH) |
| Naka Saito (2018)^34^ | Retrospective cohort | PH Patients undergoing both echocardiography and RHC. | 77 | NA | 2015 ESC/ERS | 2D-AVR+LVEF | 0.90 (Pre-PH vs. Post-PH) |
| Katleen Swinnen (2023)^35^ | Retrospective cohort | Consecutive patients with idiopathic/heritable/drug-induced PAH or PH-LHD at a Belgian university hospital (2000-2020). | 344 | 165 | 7th WSPH | Random Forest | 0.98 (Pre-PH vs. Post-PH) |
| Nobuhide Yamakawa (2022)^36^ | Retrospective cohort | Patients from a clinical database undergoing RHC or ICU monitoring with pulmonary artery catheters at a US academic medical center (2008). | 40 | NA | 7th WSPH | S3 Strength | 0.74 (Cpc-PH vs. Others) |
| Albani, Stefano (2022)^37^ | Prospective cohort | Consecutive patients undergoing both echocardiography and RHC at three European tertiary centers (Trieste, Stockholm and Pisa, 2014–2018) | 334 | 1349 | 2015 ESC/ERS | BCI | 0.63 (Ipc-PH vs. Cpc-PH) |
| Gong, Chao (2023)^38^ | Prospective cohort | Consecutive PH-LHD patients who underwent both invasive RHC and cardiac MRI within 30 days at West China Hospital (2016–2021). | 60 | NA | 2015 ESC/ERS | PTTc | 0.85 (Ipc-PH vs. Cpc-PH) |
| Present Study | Retrospective cohort | Consecutive patients suspected of having Cpc-PH who underwent RHC and echocardiography at Shanghai Pulmonary Hospital (2010–2023). | 198 | NA | 7th WSPH | PECS | 0.76 (Cpc-PH vs. Others) |
| Present Study | Retrospective cohort | Consecutive patients suspected of having pc-PH who underwent RHC and echocardiography at Shanghai Pulmonary Hospital (2010–2023). | 198 | NA | 2015 ESC/ERS | PECS | 0.73 (Cpc-PH vs. Others) |
| AUC, area under the curve; BCI, biventricular coupling index; CMR LAVi, cardiac magnetic resonance left atrial volume Index; Cpc-PH, combined post-capillary pulmonary hypertension; DC, derivation cohort; ePLAGS, echocardiographic pulmonary to left atrial global strain ratio; ePLAR, echocardiographic pulmonary to left atrial ratio; Ipc-PH, isolated post-capillary pulmonary hypertension; LV EDVi, left ventricular end‐diastolic volume index; LVEF, left ventricular ejection fraction; NPV, negative predictive value; Others, Ipc-PH or No-PH; PECS, Predictive Echocardiography Cpc-PH Score; PH, pulmonary hypertension; Post-PH, post-capillary pulmonary hypertension; Pre-PH, pre-capillary pulmonary hypertension; PTTc, corrected pulmonary transit time; VC, validation cohort; 2D-AVR, 2D-atrial volume ratio. | | | | | | | |
